# The impact of EHR and HIE on reducing avoidable admissions: controlling main differential diagnoses

**DOI:** 10.1186/1472-6947-13-49

**Published:** 2013-04-17

**Authors:** Ofir Ben-Assuli, Itamar Shabtai, Moshe Leshno

**Affiliations:** 1 Faculty of Business Administration, Ono Academic College, Zahal Street, Kiryat Ono, IL, Israel; 2 School of Economics and Management, The college of Management Academic Studies, Rishon LeZion, IL, Israel; 3 Faculty of Management, Tel-Aviv University, Ramat-Aviv, IL, Israel; 4 Faculty of Medicine, Tel-Aviv University, Ramat-Aviv, IL, Israel

**Keywords:** Medical decision analysis, Electronic health record, Health information exchange, Medical informatics, Interoperability, Health maintenance organization, IS efficiency

## Abstract

**Background:**

Many medical organizations have invested heavily in electronic health record (EHR) and health information exchange (HIE) information systems (IS) to improve medical decision-making and increase efficiency. Despite the potential interoperability advantages of such IS, physicians do not always immediately consult electronic health information, and this decision may result in decreased level of quality of care as well as unnecessary costs. This study sought to reveal the effect of EHR IS use on the physicians' admission decisions. It was hypothesizing the using EHR IS will result in more accurate and informed admission decisions, which will manifest through reduction in single-day admissions and in readmissions within seven days.

**Methods:**

This study used a track log-file analysis of a database containing 281,750 emergency department (ED) referrals in seven main hospitals in Israel. Log-files were generated by the system and provide an objective and unbiased measure of system usage, Thus allowing us to evaluate the contribution of an EHR IS, as well as an HIE network, to decision-makers (physicians). This is done by investigating whether EHR IS lead to improved medical outcomes in the EDs, which are known for their tight time constraints and overcrowding. The impact of EHR IS and HIE network was evaluated by comparing decisions on patients classified by five main differential diagnoses (DDs), made with or without viewing the patients' medical history via the EHR IS.

**Results:**

The results indicate a negative relationship between viewing medical history via EHR systems and the number of possibly redundant admissions. Among the DDs, we found information viewed most impactful for gastroenteritis, abdominal pain, and urinary tract infection in reducing readmissions within seven days, and for gastroenteritis, abdominal pain, and chest pain in reducing the single-day admissions' rate. Both indices are key quality measures in the health system. In addition, we found that interoperability (using external information provided online by health suppliers) contributed more to this reduction than local files, which are available only in the specific hospital. Thus, reducing the rate of redundant admissions by using external information produced larger odds ratios (of the β coefficients; e.g. viewing external information on patients resulted in negative associations of 27.2% regarding readmissions within seven days, and 13% for single-day admissions as compared with viewing local information on patients respectively).

**Conclusions:**

Viewing medical history via an EHR IS and using HIE network led to a reduction in the number of seven day readmissions and single-day admissions for all patients. Using external medical history may imply a more thorough patient examination that can help eliminate unnecessary admissions. Nevertheless, in most instances physicians did not view medical history at all, probably due to the limited resources available, combined with the stress of rapid turnover in ED units.

## Background

The healthcare sector world-wide has been investing heavily in integrative and interoperable medical information systems, with the aim of improving the medical decision-making process and increasing its efficiency. However, the overall contribution of information technologies to the field is not always immediately apparent [1]. But clearly, lack of information may result in a decreased level of quality of care and unnecessary costs [2].

Health information technology (HIT) and health information exchange (HIE) are increasingly viewed as key steps in overcoming the quality, safety and efficiency problems that plague U.S. health care delivery [3]. Electronic health records (EHR) and HIE networks coordinate the storage and retrieval of patient records from multiple health sources such as laboratories, other hospitals, specialized clinics, etc., thus providing vital historical medical information that is required for critical decision-making. These systems may collect two types of information- local and external - both of which can contribute to medical decisions. Local information is locally created data and reflects the integration between various units within a specific hospital, whereas external information refers to data created at different hospitals and other points of care, thus reflecting interoperability. Vest [4] found that system access was not random, and that specific patient factors increased the odds of information access. Vest’s findings show that the more a person’s data were examined, the more likely that person was to have more emergency room visits and in-patient hospitalizations.

HIE often generate log-files [5]. Log-files are actually a documentation of events occurring in the context of a software or a technological system [6]. Log-files provide an objective and unbiased measure of system usage and are recommended for evaluating health information systems (IS) [7]. See [8] for a review on health IT usability study methodologies. The log-files in the present study were based on data collected from 2004 to 2007 from seven main hospitals owned by the main health maintenance organization (HMO) in Israel.

Log files have become a standard and essential part of large applications. Log files are commonly used for the purpose of software monitoring. It is not uncommon for log-files to be continuously generated, while occupying valuable storage space, but they provide little or no value if they are never utilized to create value.

In the framework of this research, we aimed to understand the role that EHR and HIE networks can play in reducing the number of hospital readmissions and single day admissions. We examined the impact of medical information that is provided to a decision-maker in the highly stressful environment of an ED, with its complex conditions. Esquivel et al. [9], for instance, related unsatisfactory referral communication between primary care providers and specialists to the lack of attention on how the communication technology should fits with the social environment in which it is implemented. Moreover, the use of EHR has been suggested to be even more crucial during medical emergencies [10].

Second, we explored the circumstances under which this information is most important with regard to main differential diagnoses (DDs) and information from external sources (interoperability).

The main objective of this research is to assess the impact of EHR IS and HIE network on physicians' decision-making process in an ED environment by comparing the outcomes of five main DDs. We examined whether EHR and HIE network could lead to decreases in redundant readmissions within a short period since a previous discharge. This is a widely used measure [11-13], as well as stated goal that is frequently expressed by care givers, hospital administrators, and policy makers [14]. Studies have indicated that more than half of all readmission incidents could be avoided by implementing more efficient procedures [15,16].

If a patient is readmitted shortly after a hospital stay, this might indicate that the hospital discharged the patient without proper care or the right diagnosis. Alternatively, it may suggest that the patient did not follow the doctor's instructions for various reasons, for example, because the prescribed medicine was costly, or lack of home care. However, readmission after a short time period might mean that the patient has a life threatening disease and needs to go to hospice care. In any case, a high rate of readmissions is an indication that the decision making process was faulty in some way. Recent policies seek to penalize doctors for high readmission rates, an indication of hospitals’ efforts to avoid such possibly redundant procedures [17].

Hence we examined (in addition to the readmissions measurement) whether the proportion of single-day admissions fluctuates when patients' medical history is inspected via EHR IS. We assumed that there are some single-day admissions that are uncalled for, and might be prevented if a proper medical history was available. Such scales and assumptions have been used in previous studies in the field [6,18-21]. The method of using subsets of several main differential diagnoses enabled us to compare more similar groups of patients. Sox et al. [22] emphasized the importance of medical history to medical decisions. Walker et al. [23] argued that there is a relationship between viewing medical history and improved medical care performance including admission decisions.

Additionally, a limited time period of the patients, in maintain observation wards, is also possible in the physicians' decisions in an EDs. However, in our research, this period of observation was not included in the calculated admissions.

### Research question and research hypotheses

We examined the actual effect of the use of information provided by the system, locally and externally, at the point of care on the physicians' admission decisions. Our goal was to assess the way in which this information affected the process of decision-making, as well as to monitor the outcomes of decisions when EHR IS was viewed versus when it was not viewed. For this purpose we first studied the likelihood of (a) readmissions within a short period of time since an earlier discharge and (b) single day admissions, when physicians used the EHR IS compared to when physicians did not use the EHR IS. Then we compared the reduction in readmissions within seven days and single-day admissions when local information was viewed, versus when external medical information was retrieved (interoperability). Arendts et al. [24] showed that even small changes in admission rates can result in meaningful reductions in hospital occupancy and improve system capacity.

### Research hypotheses

In this section, we define the research hypotheses deriving from the research question. We aimed to discover the relationship between the use of EHR IS and the quality of medical decision- making.

We used the seven day readmission. This measure was used as an indication of the accuracy of the initial admission decision as explained in the 'Background'. The first hypothesis is therefore:

*Hypothesis 1: There is a negative relationship between viewing EHR IS and readmissions within seven days.* This hypothesis was divided into two sub-hypotheses to capture the locality of information (external vs. local information):

*Hypothesis 1a: There is a negative relationship between viewing external information* via *EHR IS and readmissions within seven days.*

*Hypothesis 1b: There is a negative relationship between viewing local information* via *EHR IS and readmissions within seven days.*

In order to control for other time period of readmissions, we tested these hypotheses with readmissions within thirty days as well.

Similar to Hypothesis 1, we used single-day admissions to assess the appropriateness of the decisions made in the ED:

*Hypothesis 2: There is a negative relationship between viewing EHR IS (including local or external information) and single-day admissions.* This hypothesis was divided into two sub-hypotheses to capture the locality of information*:*

*Hypothesis 2a: There is a negative relationship between viewing external information* via *EHR IS and single-day admissions.*

*Hypothesis 2b: There is a negative relationship between viewing local information* via *EHR IS and single-day admissionsnn.*

The logic behind hypothesis 3 was that viewing external medical history could be evidence of a more detailed examination, hence a better understanding of the patient’s condition, thus helping to avoid unnecessary admissions. Moreover, we expected medical staff to gain additional confidence after going through a patient’s medical record, thus allowing doctors to make a dismissal decision with a lower level of uncertainty.

Hypothesis 3: The effect of viewing external medical history on avoidable admissions (both readmissions and single-day admissions) will transcend that of viewing local information.

The hypotheses (above) were tested for selected frequent diagnoses: chest pain (CP), abdominal pain (AP), gastroenteritis (GE), urinary tract infection (UTI), pneumonia organism (PO). These frequent diagnoses were chosen – prior to the data-analysis – by a panel of senior physicians in cooperation with the main HMO.

## Method

### Research field and the target population

This study focused on the main health maintenance organizations (HMO) in the state of Israel, one of the world’s largest non-governmental HMOs. This HMO serves over 3.8 million customers and employs more than 9,000 physicians and 26,000 other medical staff. The HMO operates seven general hospitals (all surveyed in this research), and many community clinics.

In 2004, the HMO deployed an EHR IS (analyzed here) and established a HIE network. The EHR IS and HIE network retrieves data from many medical systems – clinical systems such as electronic medical record and administrative systems. This unique data retrieval architecture furnished a comprehensive, integrated and real time virtual patient record available at all points of care of the HMO. The system gathers historical patient clinical and administrative data from the other healthcare information systems at the HMO’s fourteen hospitals, fifteen community clinics and thousands other points of care such as labs, imaging institutions, etc. The data included patients’ demographics, chronic medication, adverse reactions, sensitivities, detailed lab and imaging results, past diagnoses, healthcare procedures etc. Although the system aimed to serve all kind of potential users - clinical and administrative staff, the physicians are the main users of the system. The log-file used for this study analysis contains documentation of the activities of physicians. The interoperable systems enable the physician at the point care to retrieve within seconds a patient’s medical historical data available from all points care of healthcare providers that connected to the HIE network.

In terms of current policies, external data are available only to the HMO insured patients. Other HMO insured patients that visit one of the main HMO hospitals will only have local files; these include all the information that relates to previous encounters and hospitalizations at this hospital.

In addition, the database for this study covered 2004 to 2007 (after the EHR IS had been integrated into all hospitals).

### The research method and statistical tools

The research method selected for this study was track log-file analysis, which incorporates various statistical tools. The log-files in this study were restricted to the main DDs presented above (CP, AP, GE, UTI and PO).

The statistical analyses were performed using SPSS version 20 (SPSS Inc., Chicago, Illinois, USA) software. Continuous variables are presented as means ± SD. To test differences in the continuous variables between the two groups, the independent samples *t*-test was performed. Associations between the dichotomous variables were tested with the Pearson Chi-square test. A multivariate logistic regression analyses was performed to test the adjusted association between the main independent variable (Viewing medical history) and the dependent variables (Readmissions and Single-Day admissions). In addition, other relevant potential cofounding variables were entered into the multivariate analysis, including age, gender, HMO, type of department, hospital and etc. The analysis considered the results in the p<0.05 level as significant.

### The dependent variables

#### Readmission within seven days

Quantified whether a patient was readmitted to a hospital within seven days from the previous discharge for a closely related condition, or otherwise. A closely related condition is defined as a condition that clinically resembles the complaint or the diagnosis which led to the previous admission.

#### Single-day admission

Quantified whether a patient, as a result of the decision to admit, was admitted for a single day or for a longer period of time. The measurement scale for single day admissions filtered out patients who intentionally sought and received treatment involving single day admission. Only admissions from an ED to a specific hospital department were recorded and included. In addition, similar to many EDs around the world, hospitals in Israel maintain observation wards in which patients are supervised for a period of 12–24 h. This period of observation was not included in the calculations.

### The independent variables

#### Viewed medical history

The patients in our study were divided into two groups: patients whose medical history was viewed via the EHR IS and patients whose medical history was not viewed via the EHR IS. The EHR IS provided full integrative information only on patients belonging to the main HMO, as specified in Table 1.

**Table 1:** Types of patient medical histories available to physicians via the EHR

**Specifics **^***a***^	**Type of medical history**
Previous encounters and hospitalizations	Encounters
Information regarding the patient's previous diagnoses	Diagnoses
A list of the permanent medications taken by the patient	Medications
Previous lab tests including blood tests, pathology history	Labs
A list of the patient's known allergies	Allergy Problems
The patient's medical record, generated by family physicians	Community Clinics
Information regarding the demography of the patient	Demography Details
A list of previous surgeries	Surgical History

Note. ^a^ Data are fully available for HMO patients, while for non-HMO patients the information was limited to the specific hospital where they were seen last.

The term 'viewed medical history' refers to access to at least one of several medical history components in the EHR IS (see Table 1). This was measured as a dichotomous variable.

#### Viewed local information

Indicates examination of medical information available within the framework of local files available in a specific hospital.

#### Viewed external information (interoperability)

Indicates the viewing of external integrative medical history, which was provided online by certain health suppliers. External information concerned only HMO patients for whom both local and external types of information were available.

### Health maintenance organization

To control for major discrepancies in the quality and the amount of medical information between the main HMO patients and other HMO patients, a dichotomous variable was created.

### Differential diagnosis

The DDs of ED referred patients were entered into the database using the WHO International Classification of Diseases (ICD/10) code. It should be noted that there could have been more than one DD per patient on a single referral. In this case we selected the main DD that was assigned by the physicians.

### ED department

This variable represented the specific type of unit where the patient was evaluated in the ED such as internal medicine or surgical.

### Hospital

This variable represented the specific hospital where the patient was evaluated.

### Patient age

Continuous variable representing the age of the patient.

### Patient gender

Male/Female.

### The ethical approval and confidence

The study was approved by the institutional review board at Tel Aviv University for meeting the requirements of the Ethics Committee. As for the data anonymity, the log files that we used for this study didn't contain any personal details on the patients including the patient id. This field was hushed before we received the file so it was anonymized.

## Results

We first present descriptive statistics regarding the distribution of ED referrals during the period of research. We then present statistical tests performed to measure the relationship between the research variables. Finally, we present the main findings regarding the track log-file analysis using multivariate regression analyses. The presented statistical analyses were consistently made on the admitted patients.

### Descriptive statistics

We present the data on referrals (consisting of admissions and discharges) from the seven hospitals chosen for the research. The log-file consisted of 281,750 samples of referrals. The names of the hospitals are not disclosed for reasons of confidentiality and privacy.

Table 2 highlights a number of key variables that are implemented in the regression analyses, by HMO. The comparison of HMOs was done for reasons of information gathering in pursuing a better understanding of the study population and not for casual conclusions.

**Table 2:** Patient characteristics: Comparison of HMO insured patients vs. other HMO insured patients (All sample and the admission subset)

**Characteristics**	**The main HMO**	**Other HMOs**	**Total study sample**
**Data on all referrals (Admissions and Discharges) – All Sample**			
**Number of Referrals**	**n = 210,568**	**n = 71,182**	**n = 281,750**
Age (years)	48.6 (Quartiles: 27.6, 51.6, 69.9)	39.2 (Quartiles: 20.5, 36.5, 55.7)	46.3 (Quartiles: 25.4, 47.8, 67.3)
Male (%)	99,951 (47.5%)	35,683 (50.1%)	135,634 (48.1%)
Referrals when EHR IS Viewed (%)	68,851 (32.7%)	18,991 (26.7%)	87,842 (31.2%)
Referrals when EHR IS Viewed [Divided by Interoperability]	Local: 58,468 (27.8%)	Local: 17,197 (24.2%)	[Local: 75,665 (26.9%)
	External: 10,383 (4.9%)	External: 1,794 (2.5%)]	External: 12,177 (4.3%)]
Admissions (%)	89,473 (42.5%)	26,246 (36.9%)	115,719 (41.1%)
**Additional Data Only on the Admissions' subset**			
**Number of Admissions**	**n = 89,473**	**n = 26,246**	**n = 115,719**
Admissions When EHR IS Was Viewed (% from All admissions)	34,313 (38.4%)	8,717 (33.2%)	43,030 (37.2%)
Admissions When EHR IS Was Not Viewed (% from All Admissions)	55,160 (61.6%)	17,529 (66.8%)	72,689 (62.8%)
Readmission within seven Days (% from Admissions)	2,830 (3.2%)	911 (3.5%)	3,741 (3.2%)
Readmission within 30 Days (% from Admissions)	6,802 (7.6%)	1,823 (6.95%)	8,625 (7.45%)
Single-Day Admissions (% from Admissions)	18,449 (20.6%)	6,859 (26.1%)	25,308 (21.9%)
Admission Period (days)	4.4 (Quartiles: 2, 3, 5)	3.8 (Quartiles: 2, 3, 4)	4.3 (Quartiles: 2, 3, 5)

Data are mean (and Quartiles) or number of subjects (proportion); all univariate comparisons were significant at 0.001. Percentages at each column relate to the column itself (the 100% for each column appears in the first line labelled "Number of Referrals" for the 'Data On all Referrals' part in the Table, and "Number of Admissions" for the 'Additional Data Only on the Admissions' subset' part of the Table).

It is clear that viewing external history did not take place very often. In terms of the entire referral population, external information was viewed in only 4.3% of the cases, compared to roughly 26.9% for viewing of local information.

It is important to note that, patients’ medical history was viewed in only 31.2% of all hospital referrals. Hence, 68.8% of all referrals did not include any use of electronic medical history.

There was greater use of medical history for patients who were members of the main HMO, for whom more extensive data had been collected (data were examined in 32.7% of the main HMO cases compared to 26.7% for the remaining patients). Yet, even among members of the main HMO, the extent of use of medical history was not exceptionally high.

### Statistical relationships between using EHR IS and the dependent variables

Tables 3 and 4 show that for most DDs, there was a substantial decrease in the rates of both readmissions and single-day admissions when EHR IS was viewed.

**Table 3:** The impact of viewing medical history on various DDs (readmissions within seven days)

**Differential diagnosis**	**Total admissions when medical history was not viewed**	**Readmissions when medical history was not viewed**	**Total admissions when medical history was viewed**	**Readmissions when medical history was viewed**	**Decrease in readmissions within seven days**	**P-Value**
All DDs	72,689 (100%)	2,956 (4.1%)	43,030 (100%)	785 (1.8%)	56.10%	<0.001
GE	5,265 (100%)	338 (6.4%)	1,900 (100%)	25 (1.3%)	79.69	<0.001
AP	14,068 (100%)	1,412 (10%)	7,511 (100%)	181 (2.4%)	76%	<0.001
CP	41,624 (100%)	865 (2.1%)	26,026 (100%)	508 (2.0%)	4.76%	p=0.257
PO	7,691 (100%)	200 (2.6%)	4,684 (100%)	50 (1.1%)	57.69%	<0.001
UTI	4,041 (100%)	141 (3.5%)	2,909 (100%)	21 (0.7%)	80%	<0.001

The percentages in the Table were calculated out of the total number of admissions. For instance, the percentage of readmissions when medical history was viewed for all DDs: 4.1% is gained as consequence of dividing the number of Readmissions when medical history was viewed (2956) with the total number of admissions in which medical history was viewed (72,689), See at Table 2. All similar percentages were calculated similarly.

*** p<0.001, ** p<0.01, *p<0.05, + p<0.1; n/a not applicable (all similar tables below use the same conventions).

**Table 4:** The impact of viewing medical history on various DDs (single-day admissions)

**Differential diagnosis**	**Total admissions when medical history was not viewed**	**Single-day admissions when medical history was not viewed**	**Total admissions when medical history was viewed**	**Single-day admissions when medical history was viewed**	**Decrease in single-day admissions**	**P-Value**
All DDs	72,689 (100%)	17,812 (24.5%)	43,030 (100%)	7,496 (17.4%)	28.98%	<0.001
GE	5,265 (100%)	1,931 (36.7%)	1,900 (100%)	428 (22.5%)	38.69%	<0.001
AP	14,068 (100%)	4,991 (35.5%)	7,511 (100%)	1,601 (21.3%)	40.00%	<0.001
CP	41,624 (100%)	9,646 (23.2%)	26,026 (100%)	4,917 (18.9%)	18.53%	<0.001
PO	7,691 (100%)	785 (10.2%)	4,684 (100%)	346 (7.4%)	27.45%	<0.001
UTI	4,041 (100%)	459 (11.4%)	2,909 (100%)	204(7%)	38.60%	<0.001

See explanations for the calculations in the footnote of Table 3.

The strongest decreases were observed for UTI, GE and AP. An exception was CP DD in which viewing of medical history had no effect on seven day readmissions.

### Results for the multiple regression analysis

The main findings regarding the track log-file analysis are summarized below. The results of each Table were analyzed separately, showing the odds ratios (OR) (of the β coefficients). Additionally, the results are adjusted to age, gender, HMO, control variables for type of ED and control variables for type of hospital. Finally, we tested all the readmissions' hypotheses also in thirty days timeframe (in addition to the seven days) and we gained similar results.

Table 5 shows that viewing external medical history via the EHR IS was consistently associated with a reduced number of seven day readmissions. When external medical history was viewed, the likelihood of seven day readmissions decreased by 74% for AP and by 69% for UTI (OR = 0.256 and 0.308 respectively). For all DDs taken together, there was a significant reduction of 48% (OR = 0.520) in seven day readmissions.

**Table 5:** Logistic regression on readmission within seven days for all DDs when viewing external medical history (Hypothesis 1a)

**Theory variables in the equation**	**All DDs ( *N *=115,719)**	**GE ( *N *=7,165)**	**AP ( *N *=21,579)**	**UTI ( *N *=6,950)**	**CP ( *N *=67,650)**	**PO ( *N *=12,375)**
External history viewed ^a^	0.520 (<0.001)	0.318 (0.051)	0.256 (<0.001)	0.308 (0.021)	0.807 (0.176)	0.867 (0.714)
Age	0.976 (<0.001)	0.998 (<0.001)	0.981 (<0.001)	0.982 (<0.001)	0.981 (<0.001)	0.971 (<0.001)
Gender ^c^	0.792 (<0.001)	1.102 (0.376)	0.638 (<0.001)	0.803 (0.222)	1.264 (<0.001)	1.138 (<0.001)
HMO ^β^	1.089 (0.031)	0.704 (0.003)	1.160 (0.012)	1.196 (0.363)	1.276 (<0.001)	1.149 (0.380)
Constant	0.123 (<0.001)	0.091 (<0.001)	0.181 (<0.001)	0.066 (<0.001)	0.047 (<0.001)	0.063 (<0.001)

The table reports a series of multiple regression analyses. Block 2 (control for type of department) and Block 3 (control for type of hospital) are not shown here, but were also included in the regression. Table entries represent the odd ratio, with p-values in parentheses. *CP* = chest pain; *AP* = abdominal pain; *GE* = gastroenteritis; *UTI* = urinary tract infection; *PO* = pneumonia organism; ^*a*^*Coded 0*= Medical history not viewed, *1*=Medical history viewed. ^*β*^*Coded 0* = other HMO, *1* = main HMO. ^*c*^*Coded 0* = female, *1*= male. (all similar tables below use the same abbreviations).

In addition, being a main HMO patient was positively correlated with higher odds for seven day readmission. This is not surprising, since the HMO owns all these hospitals, and it is likely that HMO patients will seek medical care at these hospitals more than patients belonging to other HMOs.

The results of Table 6 mirror those in Table 5. In general external medical history viewed via the EHR IS was positively and significantly correlated with reduced odds for single-day admissions. Viewing external medical history was associated with a substantial reduction of up to 35% in single-day admissions. Being a member of the main HMO was positively correlated with lower odds of single-day admission.

**Table 6:** Logistic regression on single-day admissions for all DDs for external medical history (Hypothesis 2a)

**Theory variables in the equation**	**All DDs ( *N *=115,719)**	**GE ( *N *=7,165)**	**AP ( *N *=21,579)**	**UTI ( *N *=6,950)**	**CP ( *N *=67,650)**	**PO ( *N *=12,375)**
External history viewed	0.760 (<0.001)	0.648 (0.014)	0.649 (<0.001)	0.917 (0.590)	0.844 (<0.001)	0.876 (0.445)
Age	0.979 (<0.001)	0.982 (<0.001)	0.977 (<0.001)	0.985 (<0.001)	0.968 (<0.001)	0.982 (<0.001)
Gender	0.960 (0.005)	1.004 (0.933)	0.967 (0.285)	0.904 (0.263)	0.822 (<0.001)	1.047 (0.478)
HMO	0.859 (<0.001)	0.786 (<0.001)	0.936 (0.052)	0.864 (0.132)	0.891 (<0.001)	0.962 (0.609)
Constant	1.029 (0.189)	0.986 (0.822)	1.224 (<0.001)	0.298 (<0.001)	2.547 (<0.001)	0.260 (<0.001)

Tables 7 and 8 report these same analyses but for viewing local medical history via the EHR IS (rather than external medical history).

**Table 7:** Logistic regression on readmission within seven days for all DDs when viewing local medical history (Hypothesis 1b)

**Theory variables in the equation**	**All DDs ( *N *=115,719)**	**GE ( *N *=7,165)**	**AP ( *N *=21,579)**	**UTI ( *N *=6,950)**	**CP ( *N *=67,650)**	**PO ( *N *=12,375)**
Local history viewed	0.563 (<0.001)	0.249 (<0.001)	0.276 (<0.001)	0.246 (<0.001)	1.016 (0.789)	0.567 (0.001)
Age	0.977 (<0.001)	0.991 (<0.001)	0.983 (<0.001)	0.983 (<0.001)	0.981 (<0.001)	0.972 (<0.001)
Gender	0.795 (<0.001)	1.114 (0.325)	0.642 (<0.001)	0.798 (0.211)	1.265 (<0.001)	1.149 (0.292)
HMO	1.081 (0.048)	0.697 (0.003)	1.147 (0.021)	1.172 (0.421)	1.270 (<0.001)	1.155 (0.364)
Constant	0.132 (<0.001)	0.10 (<0.001)	0.276 (<0.001)	0.079 (<0.001)	0.047 (<0.001)	0.067 (<0.001)

**Table 8:** Logistic regression on single-day admissions for all DDs explained when local medical history (Hypothesis 2b)

**Theory variables in the equation**	**All DDs ( *N *=115,719)**	**GE ( *N *=7,165)**	**AP ( *N *=21,579)**	**UTI ( *N *=6,950)**	**CP ( *N *=67,650)**	**PO ( *N *=12,375)**
Local history viewed	0.785 (<0.001)	0.776 (<0.001)	0.617 (<0.001)	0.633 (<0.001)	0.846 (<0.001)	0.887 (0.094)
Age	0.979 (<0.001)	0.983 (<0.001)	0.978 (<0.001)	0.985 (<0.001)	0.968 (<0.001)	0.982 (<0.001)
Gender	0.961 (0.007)	1.008 (0.885)	0.972 (0.364)	0.903 (0.256)	0.821 (<0.001)	1.050 (0.451)
HMO	0.855 (<0.001)	0.782 (<0.001)	0.929 (0.031)	0.865 (0.133)	0.888 (<0.001)	.962 (0.611)
Constant	1.069 (0.002)	1.014 (0.824)	1.327 (<0.001)	0.326 (<0.001)	2.625 (<0.001)	0.264 (<0.001)

Similar to the external medical history findings, local history was associated with a significant reduction in readmissions across the board, ranging from 43% for PO (OR = 0.567) to 75% for both GE and UTI (OR = 0.249 and 0.246 respectively), and 72% for AP.

Table 7 also shows that HMO correlates with readmissions rates. Moreover, being an HMO member was positively correlated readmission. These correlations were not consistent across the DDs.

Table 8 shows that viewed local history was associated with a reduced number of single-day admissions. The largest reduction was again for GE, AP, and UTI with reduction of 22% (OR = 0.776), 38% (OR = 0.617), and 37% (OR = 0.633) respectively.

Additionally, it shows a consistent association of reduced single-day admissions for patients belonging to the main HMO.

Tables 5– 8 examined external and local medical information independently. Tables 9 and 10 compare the two cases. These two regressions include a much smaller sample size (*N* = 40,030) because only cases in which information was viewed were included in the regression.

**Table 9:** Logistic regression on readmission within seven days for all DDs comparing local and external medical history (Hypothesis 3)

**Theory variables in the equation**	**All DDs ( *N *=43,030)**	**GE ( *N *=1,900)**	**AP ( *N *=7,511)**	**UTI ( *N *=2,909)**	**CP ( *N *=26,026)**	**PO ( *N *=4,684)**
Local history viewed	1.272 (0.050)	1.149 (0.482)	1.393 0(.203)	1.082 (0.894)	1.301 (0.114)	0.763 (0.526)
Age	0.989 (<0.001)	0.986 (<0.001)	0.991 (0.026)	1.003 (0.783)	0.988 (<0.001)	0.980 (0.009)
Gender	1.435 (<0.001)	1.117 (0.343)	1.092 (0.576)	1.137 (0.777)	1.587 (<0.001)	1.019 (0.947)
HMO	1.334 (0.002)	0.716 (0.016)	1.466 (0.050)	0.915 (0.877)	1.438 (0.002)	0.931 (0.884)
Constant	0.000 (0.998)	0.000 (1.000)	0.000 (0.998)	0.000 (0.999)	0.000 (0.999)	0.000 (0.999)

Note. ^*a*^*Coded 0* = external history viewed, *1*= local history viewed. (all similar tables below use the same abbreviations).

**Table 10:** Logistic regression on single-day admissions for all DDs comparing local and external medical history (Hypothesis 3)

**Theory variables in the equation**	**All DDs ( *N *=43,030)**	**GE ( *N *=1,900)**	**AP ( *N *=7,511)**	**UTI ( *N *=2,909)**	**CP ( *N *=26,026)**	**PO ( *N *=4,684)**
Local history viewed	1.130 (0.005)	1.149 (0.482)	1.024 (0.802)	0.883 (0.507)	1.109 (0.069)	1.028 (0.879)
Age	0.978 (<0.001)	0.986 (<0.001)	0.978 (<0.001)	0.989 (0.002)	0.971 (<0.001)	0.997 (0.423)
Gender	1.052 (0.066)	1.117 (0.343)	1.070 (0.266)	.856 (0.318)	0.921 (0.016)	1.183 (0.147)
HMO	0.898 (<0.001)	0.716 (0.016)	0.833 (0.006)	1.104 (0.616)	0.948 (0.177)	0.971 (0.846)
Constant	0.350 (0.031)	0.000 (1.000)	0.667 (0.478)	0.000 (0.999)	0.564 (0.649)	0.000 (0.999)

When contrasted with external medical information, viewed local medical history had a marginally significant association with an increased number of readmissions (OR = 1.272). We were not able to demonstrate this difference for the various DDs separately. We assessed that no significance was found for each DD due to small sample size. However, the direction of OR was kept solidly positive (OR range 1.149 to 1.393), except for PO DD (OR = 0.763), after controlling for all variables.

In Table 10, after contrasted with an external information, local medical history viewed had a significant association with increased number of single-day admissions (OR = 1.130) in comparison to external medical information. Again, this effect could not be found for the various DDs separately, probably due to the small sample size. Nevertheless, the direction of the association remained positive (OR range 1.024 to 1.149), except for UTI DD (OR = 0.883).

### Main findings and results

Patient medical history was only viewed in 31.2% of all referrals to the ED via the EHR. Hence, 68.8% of all referrals did not involve any use of EHR IS. There was increased use of EHR IS for patients who were members of the main HMO, for whom more extensive data were collected and available for review.

Second, viewing local and external medical history via the EHR IS was positively associated with a reduced number of readmissions within seven days (hypothesis 1 supported) and single-day admissions (hypothesis 2 supported).

Third, GE, AP, and UTI DDs were found to have the highest association between EHR IS viewed and reduced number of seven day readmissions. GE, AP, and CP DDs had the highest association between EHR IS viewed and reduced single-day admission rates.

Finally viewing external medical history was more highly correlated with lower single-day admissions and seven day readmissions than local medical history (hypothesis 3 supported).

## Discussion

Viewing medical historical information via an EHR IS and interoperable HIE networks can improve health care delivery and individual patient care in various ways. First, EHR and HIE can provide the care giver with complete, accurate, and searchable health information, available at the point of diagnosis and care, allowing for more informed decision making to enhance the quality and reliability of health care delivery. Second, they have the potential to enable more efficient and convenient delivery of care, without having to wait for the exchange of records or paperwork and without requiring unnecessary or repetitive tests or procedures and/or avoidable redundant admissions and readmissions. And third, they enhance earlier diagnosis and characterization of disease, with the potential to thereby improve outcomes and reduce costs.

This study results show that the use of EHR and HIE relates to admission decisions and constituent for the reduction in the number of seven days readmissions and single-day admissions for all patients. The results suggest that short redundant admissions and readmissions, some of which may be caused by lack of viewing or accessing medical information or by using incomplete information, can be avoided in significant percentages, by using local and external interoperable medical history from EHR IS and HIE networks during the course of assessment in the ED.

Findings also demonstrate the higher effect of using external medical history, as compared with local medical history. External medical history examination implies a more thorough patient examination that can help avoid redundant admissions. Esquivel et al. [9] proposed that flexibility in the referral process is necessary for effective system use by staff. These findings, regarding interoperability, provide insight on the benefits of adopting, implementing and using EHR IS and HIE networks in order to improve healthcare delivery by allowing more interaction with and transfer of information about patients to caregivers, and clinical care coordinators, and therefore, increasing efficiencies related to clinical and administrative tasks. We think that this study results will help decision makers in the health sector to better understand the managerial and organizational benefits and outcomes of sharing data among health care institutions.

We noted that in most instances medical history is not viewed at all, probably due to lack of complete information and the limited resources available to public medical facilities, combined with an especially emergent nature of the ED units. Therefore, in instances where medical history is available and viewed, clinical staff may gain certain confidence, thus allowing medical staff to make more effective decisions with lower uncertainty-related biases.

Therefore, we suggest that health care organizations, seeking to gain clinical, managerial and administrative benefits, may invest additional costs and efforts in health information technology in general and in EHR and HIE networks in particular. These investments will provide additional engagement of doctors with more complete, comprehensive, accurate and updated patient medical historical information, and than should produce better decision making, and in parallel eliminate unnecessary admission costs for the HMO, and therefore for its patients.

### Contribution

In the field of healthcare, physicians need information to help them provide better medical treatment, improve patients safety and more efficient medical services. One of the major issues in this context is how information on patients, supplied by EHR IS and HIE networks, under the serious time constraints and overcrowding of an ED, can improve decision-making and its outcomes. This study attempted to inform researchers, policy makers, physicians and patients by providing further insights into the field of medical informatics.

First of all, the main conclusions of this study shed light on the importance of medical history available to physicians at the point of care. Physicians take advantage of medical history, and are aware of its importance. This study specifically selected types of diagnoses where information is more important in certain cases and less so in others. Guidelines thus can be readily implemented and updated.

In addition, we illustrated the specific value of external medical information versus local data. We found a reduction in the volume of short-term admissions and single-day admissions (as compared to [6,19,20]). This reduction was substantial for both local and external medical history, with the latter making a greater contribution. This finding confirms the potential benefits of using HIE interoperable networks and thus encourages decision makers to invest more effort in improving connectivity between various healthcare entities. Third, we extend previous research by integrating DD, interoperability and patient attributes in the variables.

### Research limitations and future research

This study has some limitations. First, in this analysis we were not able to differentiate justified from unjustified admissions. We believe that many of the single-day admissions and short-term readmissions could have been avoided and more sophisticated methods of segregating avoidable and unavoidable admissions would have been helpful.

Second, the actual medical conditions of the patient were not considered in this study. These data could have helped confirm or disconfirm our claim that viewing medical history improves medical care, which in turn may reduce future costs in terms of fewer readmissions. We partially controlled this by using the subsets of main DDs.

Third, future studies could focus on exploring the impact of using different historical medical information components on admission decisions for each DD. This is particularly relevant to analyzing external data. The results will help better understand interoperability between various points of care as well as providing greater insights into ways to display shared data.

Finally, different physicians maintain different philosophies regarding the use of the system. Actual usage of the system at each of the seven hospitals was idiosyncratic because of differences in management policy relating to the system, electronic order entry in general, and the influence of other technologies on cooperation among physicians within each hospital. We suggest that future work should concentrate on adding the physicians’ attributes to the log file. Such identification was missing from this study log-file. In this context, network externalities and diffusion theory can be used to study the causes for system as well as specific information type usage. In addition, the assessment of the medical value of information could be expanded to other points of care and other departments as well.

## Abbreviations

EHR: Electronic health record; HIE: Health information exchange; HMO: Health maintenance organization; IS: Information systems; IT: Information technology; ED: Emergency department; DD: Differential diagnosis.

## Competing interests

The authors declare that they have no competing interests.

## Authors’ contributions

All contributors co-participated in an equal manner. All authors read and approved the final manuscript.

## Pre-publication history

The pre-publication history for this paper can be accessed here:

http://www.biomedcentral.com/1472-6947/13/49/prepub
